# Evaluation of Metal Tolerance of Fungal Strains Isolated from Contaminated Mining Soil of Nanjing, China

**DOI:** 10.3390/biology9120469

**Published:** 2020-12-15

**Authors:** Fiza Liaquat, Muhammad Farooq Hussain Munis, Urooj Haroon, Samiah Arif, Saddam Saqib, Wajid Zaman, Ali Raza Khan, Jianxin Shi, Shengquan Che, Qunlu Liu

**Affiliations:** 1 School of Agriculture and Biology, Shanghai Jiao Tong University, Shanghai 200240, China; fiza.liaquat@bs.qau.edu.pk (F.L.); samiaharif24@gmail.com (S.A.); 2 Department of Plant Sciences, Faculty of Biological Sciences, Quaid-i-Azam University, Islamabad 45320, Pakistan; munis@qau.edu.pk (M.F.H.M.); uharoonsheikh@gmail.com (U.H.); 3 State Key Laboratory of Systematic and Evolutionary Botany, Institute of Botany, Chinese Academy of Sciences, Beijing 100093, China; saddamsaqib.qau@gmail.com (S.S.); shangla123@gmail.com (W.Z.); 4 University of Chinese Academy of Sciences, Beijing 100049, China; 5 Zhejiang Key Lab of Crop Germplasm, Department of Agronomy, College of Agriculture and Biotechnology, Zhejiang University, Hangzhou 310058, China; alirazakhan.qau@gmail.com; 6 Joint International Research Laboratory of Metabolic and Developmental Sciences, School of Life Science and Biotechnology, Shanghai Jiao Tong University, Shanghai 200240, China; jianxin.shi@sjtu.edu.cn; 7 Department of Landscape Architecture, School of Design, Shanghai Jiao Tong University, Shanghai 200240, China; chsq@sjtu.edu.cn

**Keywords:** heavy metals, *Komagataella phaffii*, tolerance index, Cd, Cr, Pb, bioaccumulation capacity

## Abstract

**Simple Summary:**

In this study, cadmium, chromium, and lead tolerant microbes have been isolated from contaminated mining soil and characterized. Molecular characterization of isolated fungi was performed and amplified sequences were deposited in the GenBank NCBI database. Metal tolerance of the various strains has been determined by measuring the minimum inhibitory concentrations (MICs) and the tolerance indexes of all the tested strains against Cd, Cr, and Pb. Bioaccumulation capacities of *Trichoderma harzianum* and *Komagataella phaffi* have also been assessed. These findings helped us find a novel strain of *Komagataella phaffi* and suggested it to be the potential mycoremediation microbe to alleviate the contamination of Cd, Cr, and Pb. Future studies of this fungal strain can help us to understand its resistance mechanism against other heavy metals, too.

**Abstract:**

Rapidly increasing industry has resulted in greater discharge of hazardous chemicals in the soil. In the current study, soil samples were collected from Nanjing mine (32°09′19.29″ N 118°56′57.04″ E) and subjected to heavy metal analysis and microbe isolation. A total of 460 fungi were isolated, and five of these were yeast strains. Most of the strains exhibited tolerance to one metal. Five multimetal tolerant strains were selected and identified as *Aspergillus sclerotiorum*, *Aspergillus aculeatus*, *Komagataella phaffii*, *Trichoderma harzianum,* and *Aspergillus niger*. Isolated strains were grown in high concentrations of cadmium (Cd), chromium (Cr) and lead (Pb), for induced-tolerance training. The tolerance index (TI) revealed the highest Cd tolerance of novel *K. phaffii* strain at 5500 ppm (TI: 0.2). *K. phaffii* also displayed resistance at 4000 ppm against Cr (TI: 0.32) and Pb (TI: 0.32). In contrast, tolerance training for *A. niger* was not that successful. *K. phaffii* also displayed the highest bioaccumulation capacity for Cd (25.23 mg/g), Cu (21.63 mg/g), and Pb (20.63 mg/g) at 200 ppm. Scanning electron microscopy (SEM) explored the morphological changes in the mycelia of stressed fungi. Results of this study describe this delicate approach to be species and metal dependent and suggest a potential utilization of this fungal strain for the bioremediation of contaminated soils.

## 1. Introduction

Industrialization has resulted in increasing heavy metal contamination in the soil and its remediation is one of the biggest challenges of this century. Due to different industrial activities, metal pollutants are released into the soil and environment where various living organisms are being affected. Heavy metals are usually released and deposited in the soils with industrial wastes [1]. Plants need essential metals such as zinc, copper, iron, and manganese for proper growth. Non-essential metals including cadmium, lead, and mercury impose toxic effects on plant morphology [2]. Industrial soils contain high quantities of cadmium and lead which are very toxic to the environment [3]. Previously, chemical methods were adopted to remove soil toxicity such as utilizing electric fields, use of ammonium bicarbonate, and EDTA washing. [4]. Recently, researchers found that fungal species possess significant potential to absorb toxic compounds, such as heavy metals from soil, in an eco-friendly way [5,6]. 

Previously, various techniques such as precipitation and ion exchange have been applied to remove heavy metals from wastewaters [7]. These techniques have many limitations—for example, potential of chemical decline over time and not being able to remove heavy metals [8]. Moreover, removing heavy metals from soil is an even more difficult task. In this situation, biological approaches can rescue plants from heavy metals. Biological approaches include the use of living organisms such as plants, bacteria, and fungi [9]. Microorganisms absorb pollutants and they can survive under metal stress due to their innate adsorption capability [10]. In addition, the potential of microbes to oxidize various inorganic elements through oxidative phosphorylation facilitates this process. Mycoremediation refers to the utilization of lower or higher fungi for the remediation of heavy metals and it has been fascinating to researchers for the last few decades [11,12]. From an industrial perspective, fungal colonization is very important because fungi possess thermotolerant and pH tolerant extremophilic enzymes [13]. The significant difficulties in the mycoremediation of fields include the bulk production of fungal inoculum, the use of right inoculum in the soil, and the determination of appropriate transporter material [14]. Mycoremediation of Chromium (Cr) and Nickel (Ni) has been reported in many fungal species such as *Penicillium* and *Aspergillus* [15,16,17]. 

Fungal species can efficiently remove heavy metals from the soil. Scientists are working on understanding remediation mechanism of these microbes. Many studies describe that the microbes possess biosorption or bioaccumulation ability and can bind metals on their surfaces. Few studies have also described the involvement of genes such as hydrohobin in imparting metal tolerance ability to different fungi [18]. Similar bioremediation mechanisms of *Rhizopus* spp., *Penicillium* spp., *Trichoderma* spp., and *Aspergillus* spp. have been described earlier [19,20]. Through the process of biosorption, *Penicillium simplicissimum* can remove Copper (Cu) and Lead (Pb) in liquid media [21]. *Trichoderma asperellum* can tolerate Cadmium (Cd), Pb, Zinc (Zn), Cu, and Chromium (Cr) through a biosorption mechanism [20]. Some studies describe that the gradual exposure of microbes to high metal concentrations results in enhanced metal tolerance and increases their capability to eliminate heavy metals [16]. Along with other microbes, yeast is also an important ubiquitous microbe for the potential bioremediation of heavy metals [22].

The present study was designed to isolate microbes from the contaminated mine soil of the Nanjing area. This study further assessed the influence of toxic metals (Cr, Cd, and Pb) on the development of these isolates and determined their metal bioaccumulation capacities. The toxic effects of selected heavy metals, on the morphology of isolates, were observed through scanning electron microscopy (SEM).

## 2. Materials and Methods

### 2.1. Sample Collection

**Figure 1:**
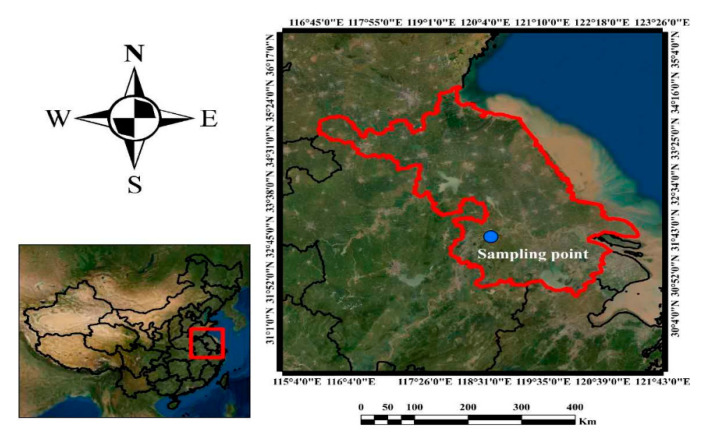
Location of studied area (Nanjing mine).

To evaluate the tolerance potential of different isolates against heavy metals (Cr, Cd, and Pb), six soil samples were collected from Nanjing mine (32°09′19.29″ N 118°56′57.04″ E) (Figure 1). Soil samples were taken from the depth of 0~30 cm and processed within 8 h. After the collection of soil samples, these were kept on dry ice and further used to isolate fungi. 

### 2.2. Processing of Soil Samples

Stones, coarse material, and other debris were removed. The air-dried soil samples were grounded into powder and sieved through a 2 mm sieve. The processed soils were used for heavy metal analysis.

### 2.3. Heavy Metal Analysis

For this analysis, 0.5 g of soil samples were weighted and transferred to beakers. A volume of 10 mL Aqua regia (HCl: HNO_3_, 3:1 *v*/*v*) was added into the soil samples to digest overnight. The beakers were gradually heated on hot plate until 1 mL of liquid remained. The extracts were filtered through Whatman filter paper and brought to volume by deionized water in volumetric flasks. After digestion, multielemental analysis of extracts was performed with an atomic absorption spectrophotometer, with a graphite furnace (Hitachi Z-8100) [23]. To evaluate the contamination level of heavy metals in the soil samples, the Pollution Index (PI), Potential Ecological Risk (RI), and Geoaccumulation Index (I_geo_) were calculated [24,25]. 

### 2.4. Fungal Isolation and Identification

The serial dilution method was used to isolate fungal strains on Potato Dextrose Agar (PDA) media. To restrict bacterial growth, streptomycin (15 mg L^−1^) and chloramphenicol (50 mg L^−1^) were added to the PDA media. The fresh soil samples (1 g each) were transferred to 100 mL of sterilized water and diluted (up to 10^−3^). In the Petri plates (90 mm), 0.1 mL of each dilution was spread and incubated at 25 °C. Petri plates (90 mm) were regularly observed, and fungal mycelia were sub-cultured on fresh PDA plates. Fungal samples were purified and stored at 4 °C. Fungal strains were identified on the basis on microscopic and molecular analyses. For the microscopic identification, slides were prepared and were observed under a light microscope. For the molecular identification, the fungal DNA was isolated using a standard protocol using 0.8% agarose gel and its quality was evaluated by using *NanoDrop* ND-1000 (*NanoDrop* Technologies Inc., Wilmington, DE, USA). For the amplification of isolated DNA, universal forward and reverse primers (ITS1F, ITS4R) were used in the C-1000 thermal cycler (Bio-Rad, Hercules, CA, USA). For PCR, 20–30 ng genomic DNA and 10 mM of each forward and reverse primer were mixed in the master mix. The cycling parameters were 94 °C for 4 min, followed by 34 cycles of 94 °C for 40 sec, 55 °C for 50 sec, 72 °C for 45 sec, and a final extension step of 72 °C for 4 min. The amplified product was subjected to Sanger’s sequencing and obtained sequences were submitted in GenBank NCBI database and subjected to BLASTn search (http://www.ncbi.nlm.nih.gov) to determine their homology. Out of all isolates, five fungi exhibited tolerance against the most occurring metals (Cd, Cr, and Pb) and were used further for the determination of the tolerance index. 

### 2.5. Tolerance Index

The tolerance towards Cd, Cr, and Pb was estimated at 2000 ppm for five fungal strains previously selected. Isolates showing tolerance at this concentration were subjected to higher range of 3000, 4000, 5000, and 6000 ppm. On the other hand, fungal isolates showing growth inhibition at 2000 ppm were further grown on reduced concentrations (100, 300, 500, 700, 1000, and 1500 ppm). To perform plate assay, fungal mycelial plugs (0.3 cm diameter) were placed on PDA media, supplemented with either salts of Cd, Cu, or Pb. PDA plates without metal salts served as control. To see fungal growth, these plates were placed at 25 ± 2 °C. The plates were photographed and fungus growth area was measured using image processing software, ImageJ 1.49 b (http://imagej.nih.gov.ij). Based on the following equation [26], the tolerance index (TI) was calculated: 

Tolerance index (TI) = fungal growth area in the presence of metal ÷ fungal growth area without metal exposure, in the same time period.

### 2.6. Bioaccumulation

Three selected toxic metals were used to validate the bioaccumulation potential of isolated fungi. Fungal strains were inoculated in Potato Dextrose Broth (PDB, Difco) and fungal biomass was collected after 7 days of incubation at 25 ± 2 °C [23]. About 0.8 ± 0.1 g of fresh biomass was transferred to 20 mL PDB, supplemented with different metal concentrations (50, 100, 150, and 200 ppm). The pH of these media was adjusted to 5 ± 0.2 and incubated at 25 ± 2 °C in a shaking incubator at 150 rpm. Metal solutions, without fungi, served as control. After one week, the samples were centrifuged to obtain cell-free supernatant. Atomic absorption spectrophotometer, with a graphite furnace (Hitachi Z-8100, Tokyo, Japan), was used for metal quantification. To measure dry weight, the remaining biomass was oven-dried and weighted. Following the method described by Simonescu and Ferdes [8], the percentage of removed metal and the amount of metal uptake were calculated. 

### 2.7. Scanning Electron Microscopy (SEM) 

Surface of fungal mycelia was observed by SEM analysis. Mycelial plugs of tolerant fungi were fixed in 2.5% glutaraldehyde and placed at 4 °C overnight. In 0.1 M phosphate buffer (pH 7.4), the samples were washed thrice. The dehydration of these samples was performed in a series of ethanol solutions (30%, 50%, 70%, 80%, 90%, 95%, and 100%). In a desiccator, samples were dried and sputter-coated with platinum (Quorum Q150RD). Morphology of these coated samples was determined by using a SEM instrument (JOEL model JED-2300). 

### 2.8. Statistical Analysis

For all experiments, triplicates were prepared. All the data obtained were subjected separately to a one-way analysis of variance (ANOVA) using the Statistical Package for the Social Sciences (SPSS) version 20.0. Means were compared with independent *t*-test (α < 0.05) and Tukey’s honestly significant difference test (*p* < 0.05).

## 3. Results

### 3.1. Heavy Metal Analysis

High concentrations of toxic metals were detected in all soil samples. The pollution levels of soil samples evaluated by PI, RI and I_geo_ values are presented (Table 1). 

### 3.2. Isolation of Microbes

A total of 460 fungal isolates—five of which were yeast strains—were isolated from the soil samples. Most of the strains exhibited tolerance against one metal only. The five identified multitolerant metal strains—*Aspergillus sclerotiorum* (E1), *Aspergillus aculeatus* (A1), *Komagataella phaffii* (WS), *Trichoderma harzianum* (Y1) and *Aspergillus niger* (G03)—were selected for further experimentation (Table 2). The identification of these fungi was accomplished on the basis of their morphological characteristics and the sequencing of their ITS-rDNA region. The obtained sequences were aligned and deposited in the GenBank NCBI database (Table 2). *Aspergillus* was the most frequently isolated species from all samples. The highest fungal diversity was obtained in soil sample 2. This sample showed low heavy metal contamination. Soil sample 4 exhibited the highest metal contamination and very few microbes could be isolated from it (Table 1).

### 3.3. Tolerance Index (TI) 

**Figure 2:**
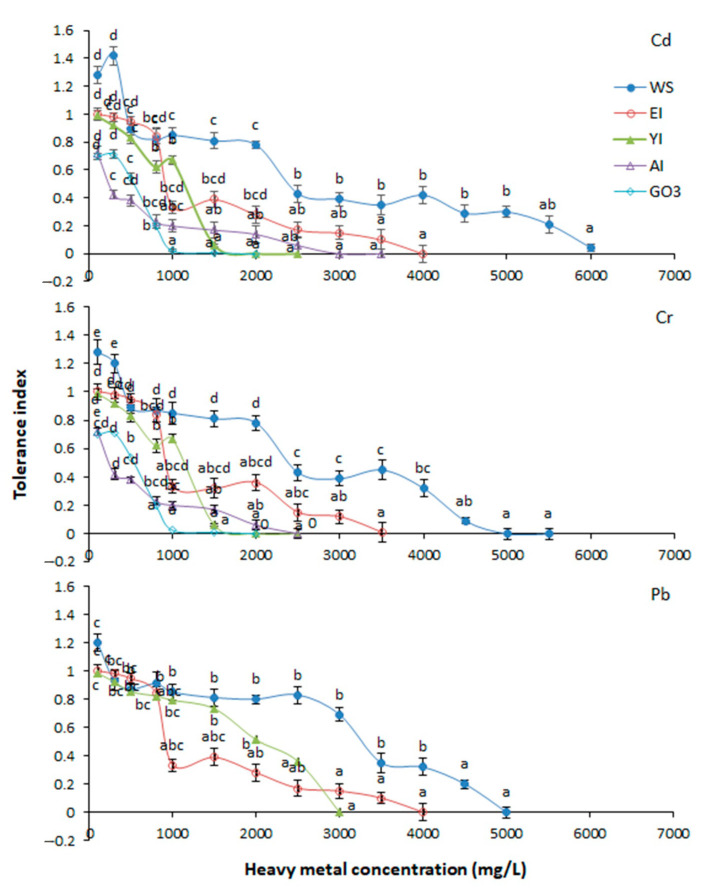
Tolerance index of fungi strains under various heavy metal concentrations. Columns denote the mean values and bars indicates standard error. Different letters show statistically significant different values (*p* < 0.05) from each other as calculated by Tukey’s honestly significant difference test. Means with same letters within the isolate groups are not significantly different at Honestly Significant Difference (HSD_(0.05)_) *Aspergillus sclerotiorum* (A1), *Aspergillus aculeatus* (E1), *Aspergillus niger* (G03), *Komagataella phaffi* (WS), and *Trichoderma harzianum* (Y1).

The five tested isolates showed different growth and tolerance capacities at varying concentration of heavy metals. The Cd was toxic towards G03 at 1000 ppm and resulted in severe growth inhibition (TI: 0.024). At the same concentration, the remaining four strains showed higher tolerance to Cd. The maximum Cd tolerance was exhibited by WS strain at 5500 ppm (TI: 0.2). Similar performance of WS was observed at 4000 for Cr (TI: 0.32) and Pb (ppm TI: 0.32) (Figure 2). These results indicated significant growth response of WS in Cd, Cr, and Pb contamination. The highest Cr tolerance was observed by E1 isolate at 3000 ppm (TI: 0.12), followed by A1 at 1500 ppm (TI: 0.17), and Y1 and G03 at 1000 ppm (TI: 0.024–0.67). On the contrary, Y1 demonstrated better tolerance to Pb at 2500 ppm (TI: 0.36). Exceptionally, at a lower concentration for each metal, TI values of WS were higher than 1, which means that the WS strain thrives with Cd (100–300 ppm, TI: 1.28–1.42), Cr (100–300 ppm, TI: 1.2–1.28), and Pb (100 ppm, TI: 1.2) at low concentrations. All the selected fungal strains were able to tolerate 1500 ppm of various metals. So, the TI of the selected fungal strains was compared under 1500 ppm of Cd, Cr, and Pb. The results indicated that the highest tolerance capacity for all the three metals was exhibited by WS. 

### 3.4. Morphological Changes

**Figure 3:**
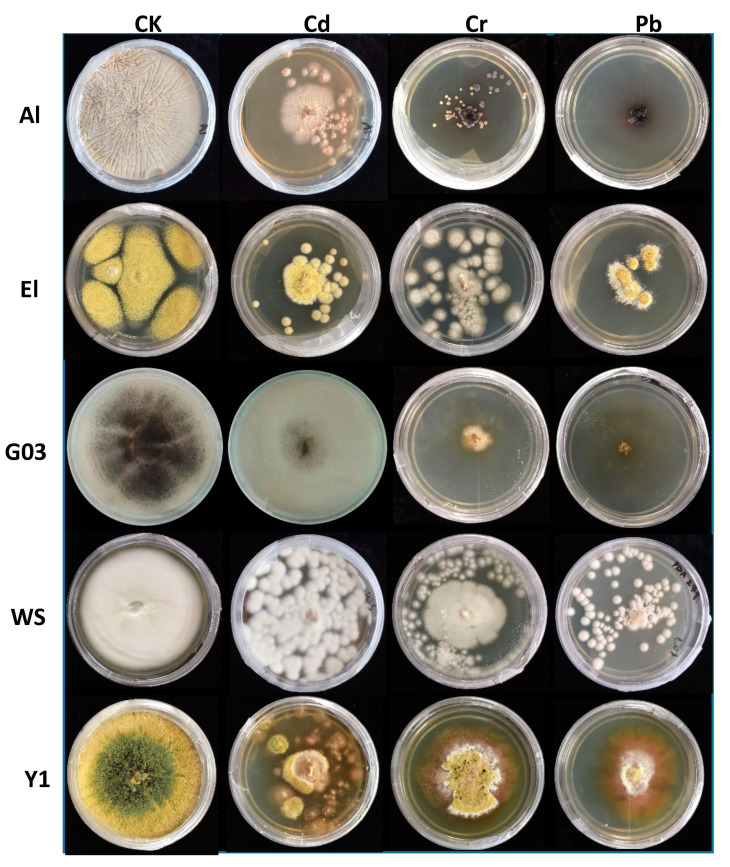
Growth of fungi on media supplemented with 1500 ppm concentration of Cd, Cr, and Pb. Five isolates including *Aspergillus sclerotiorum* (A1), *Aspergillus aculeatus* (E1), *Aspergillus niger* (G03), *Komagataella phaffi* (WS), and *Trichoderma harzianum* (Y1) were grown on control media (CK), cadmium supplements media (Cd), chromium supplemented media (Cr), and lead supplemented media (Pb). The growth of *Aspergillus sclerotiorum* (A1) and *Aspergillus niger* (G03) was observed to be severely retarded in Pb amended media.

In this study, upon metal exposure, changes in the morphology of fungal colonies were observed and these changes were more prominent at higher metal concentrations (Figure 3). Strain A1 showed small and separated colonies on media supplemented with Cd, while these colonies were dark brown and white in Pb supplemented with control media, respectively. The presence of Cr resulted in whitish and smaller colony growth of E1, but under the influence of Cd and Pb, smaller and more separated colonies of E1 could be observed. In the controlled condition, E1 colony was bigger than all other treatments. In the presence of Cr and Pb, strain G03 demonstrated severe growth inhibition and formed a brown colony in the center with brownish-white mycelia. Under the exposure of Cd, the color of G03 did not change much but showed strong growth inhibition. In the presence of metals, WS formed little spot-shaped greasy white colonies instead of a plain white mass. In the presence of metals, Y1 formed white colonies. Moreover, in all metal supplemented media, orange color pigmentation, on the edges of colony mycelia, was also observed. 

### 3.5. Bioaccumulation Capacity

**Figure 4:**
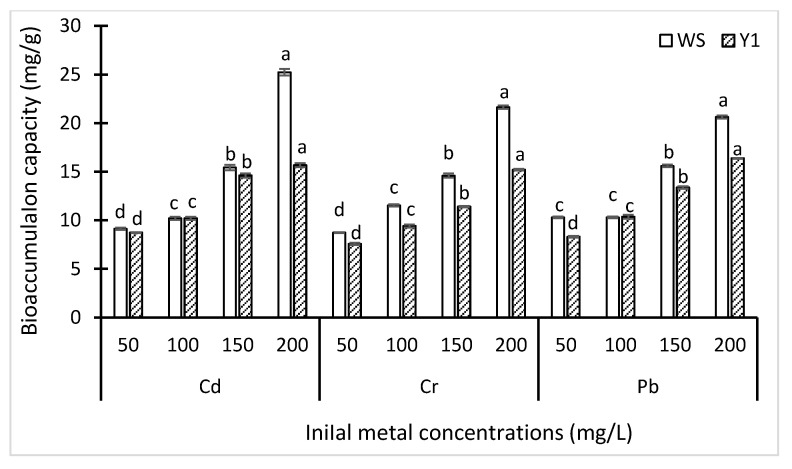
Influence of initial concentrations of Cd, Cu, and Pb on bioaccumulation capacities of five cells of *Komagataella phaffi* (WS) and *Trichoderma harzianum* (Y1). Bars indicate standard error of mean (±SE). Means values with same letters are not significantly different, calculated by Tukey’s honestly significant difference test.

Variable bioaccumulation capacities of two strains (WS and Y1) were observed under different metal concentrations (Figure 4). Generally, the bioaccumulation capacity of WS was better than Y1. These two isolates showed better capacity with the increasing concentration of each metal. The highest bioaccumulation capacity was demonstrated by WS at 200 ppm of Cd (25.23 mg/g). Similarly, the capacity of WS was slightly higher for Cu (21.63 mg/g) than Pb (20.63 mg/g) at 200 ppm. On the contrary, Y1 demonstrated the highest bioaccumulation capacity at 200 ppm of Pb (16.37 mg/g). 

### 3.6. Scanning Electron Microscopy

**Figure 5:**
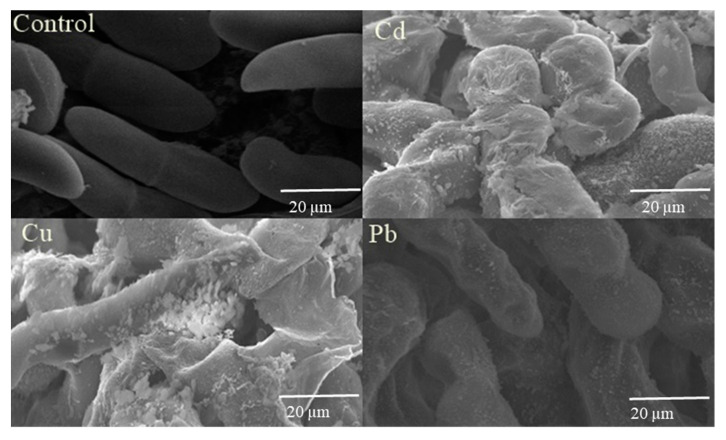
Scanning electron microscopy (SEM) photomicrographs of *K. phaffi* (WS), grown with no metal (control), with 4000 ppm cadmium (Cd), with 4000 ppm chromium (Cr) and with 4000 ppm lead (Pb).

Scanning electron microscopy (SEM) analysis described the deposition of Cd, Cr, and Pb on the cell surface of the WS strain. Irregularly shaped mycelia of the WS strain could be observed in SEM images while these were healthy and intact in control. Interestingly, this particular isolate produced extracellular material that could possibly adsorb the metal onto its surface (Figure 5).

## 4. Discussion

Awareness about environmental pollution has developed regulatory processes that intend to decontaminate the environment. For the management of specific contaminants, each developed technology has its benefits and drawbacks. Bioremediation and phytoremediation in connection with microorganisms are modern technologies to solve various environmental pollution problems. Due to its major existence in polluted sites, the application of fungi in bioremediation is well documented [27].

In this study, high levels of toxic metals were found in the industrial soil samples, collected from Nanjing mine, China. The aim of this study was to isolate metal tolerant fungal strains. Out of 460 strains, five multitolerant strains including *Aspergillus sclerotiorum*, *Aspergillus aculeatus*, *Komagataella phaffi Trichoderma harzianum,* and *Aspergillus niger* were selected for further experimentation and removal of heavy metals from the collected soil samples. Heavy metals do not degrade naturally, so their removal from the soil has become a great challenge in the modern era [28]. Use of microbes is one of the possible solutions to remove heavy metals from contaminated soil. Due to their natural adsorbent ability, they can up take metals and clean the soil [29].

The selected strains showed varied tolerance capacities in different concentrations of metals. All the selected fungal strains were able to tolerate 1500 ppm of various metals. *Aspergillus* and *Trichoderma* have been reported to tolerate Pb and Cu [21]. Many studies have described the isolation of *Aspergillus*, *Penicillium,* and *Fusarium* from metal stress conditions [30,31]. These microbes can efficiently remove heavy metals [28,32,33,34]. *Trichoderma* species have also showed good tolerance against lead, zinc, copper, nickel, and cadmium [35]. *Aspergillus aculeatus* have the ability to tolerate Cd stress [36]. *Trichoderma* fungal species has great potential of bioremediation [37]. The TI values of *K. phaffi* were higher than 1 so it is one of the potential candidates that exhibited maximum tolerance to Cd, Cr, and Pb. Previous studies also described the use of *Pichia pastoris* for the uptake of silver and selenium and biotransformation. Cytochrome-b5 reductase helps *Pichia* to survive in high heavy metal concentrations [38]. *P. pastoris* also has the ability to accumulate copper [39].

Under metal exposure, clear change in colony morphology was observed which was more prominent in the presence of higher metal concentrations. It has been reported earlier that the production of pigments indicates the precipitation of metal ions on the fungal cell wall [40]. However, the change in color of the fungal cell wall is probably due to metal sequestration [41].

The better bioaccumulation capacity was exhibited by *K. phaffi.* However, the bioaccumulation capacity increases with an increase in the initial metal concentration. The bioaccumulation capacities of *K. phaffi* for Cd, Cu, and Pb were found to be 25.23, 21.63 and 20.63mg/g. These findings are in accordance with previous studies [42], in which it was observed that with the increase in initial metal concentration, the bioaccumulation capacity of fungi also increased. This is the first study reporting on the bioaccumulation capacity of *K. phaffi* for metals (Cd, Cu and Pb) in aqueous solutions.

Scanning electron micrographs revealed the deposition of Cd, Cr, and Pb on the cell surface of *K. phaffi*. Due to the accumulation of metals, the mycelia of *K. phaffi* was observed to be irregular in shape. Our findings are similar to the findings of [43] who observed the similar morphological changes under metal exposure.

## 5. Conclusions

The present study showed very high metal contamination in the soil of the Nanjing mining area. Among the isolated species from the soil, *T. harzianum* (Y1, MT229305) and *K. phaffii* (WS, MT229304) showed enhanced tolerance towards increasing concentrations of heavy metals (Cd, Cr, and Pb), in induced-tolerance training. *A. niger* (G03) was less adaptable towards all three metals. A bioaccumulation mechanism was demonstrated by *T. harzianum* and *K. phaffii* to remove Cd, Cr, and Pb and a better bioaccumulation capacity was exhibited by *K. phaffi.* The maximum bioaccumulation capacity of *K. phaffii* was observed for Cd, followed by Cr and Pb. Conclusive findings of this work demonstrated high metal tolerance and bioaccumulation capacity of the novel *K. phaffii* strain and proved it a promising mycoremediation agent.

## Figures and Tables

**Table 1:** Concentrations of heavy metals (mg kg^−1^) in soil and pollution evaluation.

	Cd	Cu	Pb	Zn	Cr
Range	20.99~39.80	9.88~22.40	640.00~895.00	1098.00~2689.30	300.00~330.00
Mean	30.57 ± 7.06	15.84 ± 4.64	789.1 ± 116.22	1964.98 ± 633.22	308.83 ± 12.97
PI	28.33 ± 27.60	1.03 ± 0.04	0.03 ± 0.02	1.59 ± 0.41	3.96 ± 3.94
RI	4473.16 ± 4357.80	10.47 ± 0.42	1.93 ± 1.51	159.78 ± 41.52	25.81 ± 25.68
I_geo_	6.64 ± 6.60	1.80 ± 2.82	−1.96 ± −2.32	4.41 ± 2.47	4.11 ± 4.10

**Table 2:** Accession numbers of the isolates.

Strain No.	Fungal Species Names	Accession Number
E1	*Aspergillus sclerotiorum*	MT229298
A1	*Aspergillus aculeatus*	MT229301
WS	*Komagataella phaffi*	MT229304
Y1	*Trichoderma harzianum*	MT229305
G03	*Aspergillus niger*	MT304812
